# Serum endocan, asymmetric dimethylarginine and lipid profile in children with familial Mediterranean fever

**DOI:** 10.1038/s41390-024-03093-8

**Published:** 2024-02-23

**Authors:** Rania S. El Zayat, Fahima M. Hassan, Noran T. Aboelkhair, Walaa F. Abdelhakeem, Ahmed S. Abo Hola

**Affiliations:** 1 Department of Pediatrics, Faculty of Medicine, Menoufia University, Shebin El-Kom, Egypt; 2 Department of Clinical Pathology, Faculty of Medicine, Menoufia University, Shebin El-Kom, Egypt; 3 Egyptian Ministry of Health, Cairo, Egypt

## Abstract

**Background:**

Familial Mediterranean fever (FMF) is a chronic inflammatory disease, and it is thought that subclinical inflammation persists even when there are no attacks, eventually causing endothelial dysfunction (ED) and atherosclerosis. Limited data are available about serum endocan, asymmetric dimethylarginine (ADMA) and lipid profile in children with FMF, so we aimed to evaluate these markers in children with FMF during the attack-free period.

**Methods:**

A total of 50 patients diagnosed with FMF and 50 age and sex-matched healthy children were recruited. Serum endocan, ADMA and lipid profiles were measured. Also, atherogenic indices (Castelli’s risk indices I and II [CRI I and II], atherogenic index of plasma [AIP] and atherogenic coefficient [AC]) were calculated.

**Results:**

Serum endocan, ADMA levels, low-density lipoprotein cholesterol, triglycerides, CRI II and AIP of the FMF patients were significantly higher than controls (*p* < 0.001). Unlike serum endocan, serum ADMA showed a positive significant correlation with total cholesterol, non-high density lipoprotein cholesterol, CRI I, AIP and AC (*p* < 0.001, *p* < 0.001, *p* = 0.004, *p* = 0.028, *p* = 0.004 respectively).

**Conclusion:**

Serum ADMA and lipid profile might be used as potential markers for endothelial dysfunction and increased cardiovascular risk in FMF patients.

**Impact:**

- Theoretically, serum ADMA may affect lipid profiles and serum endocan represents an intriguing biomarker related to inflammation. Coexistence of dyslipidemia represents an additional risk factor that contributes to the onset of early atherosclerosis.
- A few studies investigated the role of changes in lipid profile and lipid ratios in accelerated atherosclerosis pathogenesis in FMF patients.
- The relationship between colchicine and lipid profile is contradictory. Although colchicine can cause dyslipidemia, it also has anti-atherosclerosis effects.
- Elevated ADMA level and atherogenic indices in FMF children reflect their potential role in the early detection of cardiovascular affection in FMF patients.

## Introduction

Familial Mediterranean Fever (FMF) is an auto inflammatory disease characterized by periodic self-limiting febrile attacks and polyserositis associated with elevated acute inflammatory markers and caused by mutations in Mediterranean fever gene (MEFV).^1^ These mutations are thought to cause uncontrolled neutrophil activation and inflammation by changing the pyrin protein inflammasome.^2^

Patients with inflammatory diseases including those with FMF are more likely to develop cardiovascular events due to the possibility for chronic persistent subclinical inflammation even during attack-free periods causing endothelial dysfunction (ED), neutrophil dysfunction, and prothrombotic conditions that may affect arterial wall extensibility, flexibility, and elasticity.^3^ In addition, dyslipidemia can coexist with auto inflammatory disorders and is one of the main risk factors for the development of future cardiovascular events. Therefore, monitoring lipid profiles is crucial for treating patients with high cardiovascular risk.^4^

Endocan, a dermatan sulfate proteoglycan molecule, is known as a particular endothelium molecule. Its production is stimulated by several inflammatory cytokines and has been observed to rise in various inflammation-related disorders.^5,6^ In addition, asymmetric dimethylarginine (ADMA) derives from vascular endothelium by irreversible posttranslational methylation of guanidino-nitrogens of arginine residues and influences variable atherogenic processes such as monocyte adhesiveness, proinflammatory and chemotactic factors expression, and accumulation of oxidatively modified low-density lipoprotein (LDL) in macrophages.^7^

Since vascular endothelial cells are believed to be a major source of endocan and ADMA, we aimed to compare serum levels of endocan, ADMA, and lipid profile in children with FMF during attack-free period to those of healthy controls.

## Methods

This study was approved by the ethical committee of the Faculty of Medicine, Menoufia University (ID 2/22PEDI55). From Pediatric Immunology and Rheumatology Clinics of Menoufia University Hospitals and between March 2022 and March 2023, a total of 50 children diagnosed with FMF according to Euro fever/PRINTO clinical plus genetic criteria,^8^ in addition to 50 age and sex-matched healthy children were enrolled in this study. Patients previously diagnosed with any cardiovascular disease, hepatic, or renal impairment, chronic auto inflammatory disease other than FMF, malignancies, hypothyroidism, hyperlipidemia, and those using drugs other than colchicine were excluded from the study.

All patients were subjected to a detailed clinical history including the age of onset of the disease, symptoms (recurrent fever, recurrent abdominal pain, and recurrent joint pain), type and dose of treatment, thorough clinical examination, and MEFV gene mutation analysis.

During an attack-free period (defined as being free of attack for at least 3 weeks.^7^), venous blood samples were withdrawn and under aseptic conditions, serum was separated for immediate assay of lipid profile [triglycerides (TG), total cholesterol (TC), LDL cholesterol (LDL-C) and high-density lipoprotein cholesterol (HDL-C)] by using AU 680 Beckmann autoanalyzer (Beckmann). C reactive protein (CRP) and serum amyloid A (SAA) were also measured, and an aliquot of serum was stored at −20 °C until serum endocan and asymmetric dimethyl arginine were estimated by enzyme linked immunosorbent assay (ELISA) according to manufacturer’s protocol (SunRed, China).

Then, atherogenic indices including Castelli’s risk index I (TC/HDL-C), CRI II (LDL-C/HDL-C), the atherogenic index of plasma “AIP” (Log TG/ HDL-C), non-HDL-C (TC-HDL-C), and the atherogenic coefficient “AC” (Non-HDL-C/HDL-C) were calculated from lipid profile results.^9–12^

### Sample size estimation

According to a review of an earlier study.^5^ and with a 10% dropout rate, the sample size was determined to be 33 subjects by using statistics and the Sample Size Pro tool version 6. As the power of study was 80% and confidence level was 95%, the sample size was increased to 50 subjects.

### Statistical analysis

Normally distributed quantitative data was presented as mean ± standard deviation (SD), while non-normally distributed quantitative data was presented as median (interquartile range ‘IQR’). The Mann-Whitney U test was used for non-normally distributed variables while t-test was used to compare the means of normally distributed quantitative data. The Kruskal–Wallis test or one-way ANOVA was used for comparing two or more independent samples of equal or different sample sizes. Qualitative data was presented as number (percent). The Chi square test was used to evaluate the association between qualitative variables. Correlations between analyzed data were tested by Spearman correlation. For all the data analyzed, a *p* < 0.05 was considered statistically significant. Receiver Operating Characteristic (ROC) curve was used to assess the diagnostic performance of serum endocan and ADMA with a higher area under the curve (AUC) indicating better performance. All the analysis was performed using IBM SPSS Statistics for Windows, Version 20.0. (IBM Corp, Armonk, NY).

## Results

There was no statistically significant difference between patients and controls regarding their age (6.47 ± 3.02 vs 6.50 ± 2.81) and sex (males 56%, females 44% vs 42% and 58%) respectively. Mean age of diagnosis in patients was 4.37 ± 1.49 years with mean duration of disease 3.66 ± 2.21 years. Regarding major symptoms for FMF diagnosis, fever was documented in 90% of patients followed by abdominal pain in 40% then arthritis in 4%. MEFV gene mutations were observed in 43 out of 50 patients (86%), in whom heterozygosity represented 90.7% (39 patients). Clinical criteria of studied patients were presented in Table (1).

**Table 1:** Demographic data of studied patients.

	No = 50	%
Sex		
Male	28	56
Female	22	44
Age of diagnoses (years)		
Min.–Max.	2.0–7.50	
Mean ± SD.	4.37 ± 1.49	
Median (IQR)	4.0 (3.0–5.0)	
Disease duration (years)		
Min.–Max.	0.50–12.0	
Mean ± SD.	3.66 ± 2.21	
Median (IQR)	3.0(2.0–5.0)	
Major Symptoms of diagnosis^a^		
Fever	45	90
Arthritis	2	4
Abdominal pain	20	40
MEFV gene mutation analysis		
Homozygous affection	4	8
Heterozygous affection	39	78
Negative	7	14

*SD* standard deviation, *IQR* Inter quartile range.

^a^Patients may present with one or more major symptoms.

M694I and E148Q were the most common mutations observed in our patients (both 23.26%). Followed by A744S mutation (20.93%) as shown in Table (2).

**Table 2:** Familial Mediterranean fever gene mutation distribution among genetically affected patients (*n* = 43).

Gene pattern	Homozygous	Heterozygous	Total
M6941	2 (4.66%)	8 (18.60%)	10 (23.26%)
E148Q	1 (2.33%)	9 (20.93%)	10 (23.26%)
A744S	0	9 (20.93%)	9 (20.93%)
M6801	0	6 (13.95%)	6 (13.95%)
V726A	1 (2.33%)	3 (6.97%)	4 (9.30%)
P369S	0	3 (6.97%)	3 (6.97%)
R202Q	0	1 (2.32%)	1 (2.32%)

As shown in Table (3), a statistically high significance was found in patients versus controls regarding serum TG and LDL-C, *p* < 0.001. Moreover, patients had a significantly increased Castelli’s risk index II (CRI II) and AIP compared to controls, *p* = 0.008 and *p* < 0.001 respectively. Serum endocan and serum ADMA levels were found to be significantly higher in the patient’s group versus the control group, *p* < 0.001.

**Table 3:** Comparison of clinical and laboratory data between patients and controls.

	Patients (*n* = 50)	Control (*n* = 50)	*p*
Clinical data			
Weight (kg) mean ± SD	22.21 ± 9.18	22.90 ± 9.24	0.761
Height (cm) mean ± SD	115.52 ± 18.39	117.50 ± 18.01	0.588
Body Mass Index (kg/m^2^) mean ± SD	16.0 ± 1.17	15.90 ± 1.88	0.772
Systolic BP (mmHg) mean ± SD	99.20 ± 7.65	97.40 ± 10.01	0.315
Diastolic BP (mmHg) mean ± SD	64.40 ± 5.12	62.60 ± 6.08	0.112
Laboratory data			
Hemoglobin (gm/dL) mean ± SD	11.54 ± 1.01	11.52 ± 1.23	0.934
White blood cell count (×10^9^/L) mean ± SD	8.0 (6.0–10.80)	7.0 (5.50–9.20)	0.099
Platelets count (×10^9^/L) mean ± SD	335.98 ± 107.71	325.04 ± 75.39	0.558
Urea (mg/dL) mean ± SD	26.32 ± 8.07	26.52 ± 7.89	0.901
Creatinine (mg/dL) median (IQR)	0.51 (0.40–0.64)	0.55 (0.50–0.68)	0.429
LDL-C (mg/dL) mean ± SD	94.98 ± 18.88	81.0 ± 13.78	<0.001^**^
HDL-C (mg/dL) mean ± SD	44.44 ± 6.98	43.18 ± 6.82	0.364
Triglycerides (mg/dL) mean ± SD	127.98 ± 44.56	90.20 ± 19.79	<0.001^**^
Total Cholesterol (mg/dL) mean ± SD	162.96 ± 18.12	158.86 ± 9.22	0.158
non-HDL-C (TC-HDL) (mg/dL) mean ± SD	118.5 ± 21.37	115.7 ± 11.04	0.406
Castelli’s risk index I (TC/HDL-C) mean ± SD	3.77 ± 0.81	3.76 ± 0.58	0.929
Castelli’s risk index II (LDL-C/HDL-C) mean ± SD	2.22 ± 0.66	1.92 ± 0.42	0.008^*^
Atherogenic index of plasma (log TG/HDL-C) mean ± SD	0.44 ± 0.17	0.31 ± 0.12	<0.001^**^
Atherogenic coefficient (non-HDL-C/HDL-C) mean ± SD	2.77 ± 0.81	2.76 ± 0.58	0.929
Serum endocan (pg/ml) median (IQR)	640.0 (350.0–800.0)	48.50 (30.0–56.0)	<0.001^**^
Serum asymmetric dimethyl arginine (ng/ml) median (IQR)	725.0 (650.0–800.0)	44.50 (33.0–60.0)	<0.001^**^

*HDL-C* high-density lipoprotein cholesterol, *IQR* inter quartile range, *LDL-C* low-density lipoprotein cholesterol, *non-HDL* non-high-density lipoprotein cholesterol, *p* p value, *SD* standard deviation.

*Statistically significant at *p* ≤ 0.05. ^**^highly Statistically significant *p* < 0.001.

Regarding mutation types in patients, we found that LDL-C and TG were significantly elevated in those with homozygous mutations when compared to those with heterozygous or no mutations (*p* = 0.006 and 0.017 respectively), On the other hand serum endocan and ADMA levels showed no significance Table (4).

**Table 4:** Comparison of lipid profile, serum endocan and asymmetric dimethyl arginine among different mutation types in patients’ group.

	FMF gene			Test of Sig.	*p*
	No mutation (*n* = 7)	Heterozygous mutation (*n* = 39)	Homozygous mutation (*n* = 4)		
LDL-C (mg/dL) Mean ± SD.	81.0 ± 16.84	95.18 ± 17.84	117.50 ± 9.57	F = 5.674*	0.006^*^
HDL-C (mg/dL) Mean ± SD.	46.29 ± 6.95	44.72 ± 7.01	38.50 ± 4.51	F = 1.778	0.180
Triglycerides (mg/dL) Mean ± SD.	125.0 ± 23.09	122.41 ± 39.46	187.5 ± 80.57	F = 4.435*	0.017^*^
Total Cholesterol (mg/dL) Mean ± SD.	157.6 ± 21.44	162.8 ± 17.31	173.8 ± 20.56	F = 1.021	0.368
Serum endocan (pg/ml) Median (Min.–Max.)	750 (80–950)	595.0 (80 –1000)	490 (100–770)	H = 2.408	0.300
Asymmetric dimethyl arginine (ng/ml) Median (Min.–Max.)	740.0 (150–900)	725.0 (110–1000)	687.5 (450–800)	H = 0.417	0.812

*SD* standard deviation, *p* *p* value * Statistically significant at *p* ≤ 0.05, *F* F for One way ANOVA test, *H* H for Kruskal–Wallis test.

Table (5) demonstrated correlation between serum ADMA, serum endocan with clinical and laboratory data in patient’s group. We found a positive significant correlation between ADMA and serum TC, non-HDL-C, Castelli’s risk index I (CRI I), AIP and AC (*p* < 0.001, *p* < 0.001, *p* = 0.004, *p* = 0.028, *p* = 0.004 respectively), conversely no correlation was found between serum endocan and any of clinical or laboratory variables.

**Table 5:** Correlation between serum endocan and asymmetric dimethyl arginine and variable clinical and laboratory data in patients.

	Serum endocan		Asymmetric dimethyl arginine	
	*r*_s_	*p*	*r*_s_	*p*
Weight (kg)	−0.162	0.260	0.224	0.117
Body Mass Index (kg/m^2^)	−0.183	0.204	−0.039	0.788
CRP in attack-free period (mg/dL)	−0.096	0.696	−0.269	0.266
Serum amyloid A in attack−free period (mg/L)	−0.075	0.603	0.188	0.192
Platelet count (×10^9^/L)	0.060	0.678	−0.068	0.639
LDL-C (mg/dL)	−0.101	0.484	0.155	0.284
HDL-C (mg/dL)	−0.024	0.870	−0.243	0.089
Triglycerides (mg/dL)	−0.106	0.465	0.263	0.065
Total Cholesterol (mg/dL)	0.034	0.813	0.487	<0.001^**^
non−HDL-C (TC-HDL) (mg/dL)	0.039	0.789	0.504	<0.001^**^
Castelli’s risk index I (TC/HDL-C)	0.027	0.854	0.402	0.004^*^
Castelli’s risk index II (LDL-C/HDL-C)	−0.088	0.545	0.214	0.135
Atherogenic index of plasma (log TG/HDL-C)	−0.058	0.690	0.311	0.028^*^
Atherogenic coefficient (non-HDL-C/HDL-C)	0.027	0.854	0.402	0.004^*^
Disease duration (years)	−0.144	0.317	0.261	0.067
Treatment duration (years)	−0.085	0.555	0.245	0.086

*r*_s_ Spearman coefficient.

*Statistically significant at *p* ≤ 0.05, **highly Statistically significant *p* < 0.001.

Linear regression analysis showed a significant association between TC, non-HDL-C, CRI I, and AIP and ADMA levels in univariate analysis, however none of them was an independent influencer for ADMA levels in multivariate analysis Table (6).

**Table 6:** Univariate and multivariate linear regression analysis for the parameters affecting asymmetric dimethyl arginine in patients.

	Univariate		^#^Multivariate	
	*p*	B (LL–UL 95%C.I)	*p*	B (LL–UL 95%C.I)
Weight (kg)	0.364	2.990 (−3.57–9.55)		
Serum amyloid A in attack-free period (mg/L)	0.425	1.678 (−2.512–5.869)		
HDL-C (mg/dL)	0.098	−7.101 (−15.56–1.36)		
Triglycerides (mg/dL)	0.100	1.107 (−0.22–2.43)		
Total Cholesterol (mg/dL)	0.008^*^	4.296 (1.18–7.41)	0.803	4.014 (−28.16–36.19)
non-HDL-C (TC-HDL) (mg/dL)	0.005^*^	3.846 (1.23–6.46)	0.956	−1.193 (−44.74–42.35)
Castelli’s risk index I (TC/HDL-C)	0.009^*^	94.903 (24.83–164.98)	0.916	29.184 (−522.72–581.09)
Atherogenic index of plasma (log TG/HDL-C)	0.033^*^	376.306 (31.23–721.38)	0.379	211.583 (−267.94–691.11)
Disease duration (years)	0.358	12.601 (−14.69–39.90)		
Treatment duration (years)	0.365	11.710 (−14.04–37.46)		

B: Unstandardized Coefficients.

*C.I* confidence interval. *LL* lower limit, *UL* upper Limit.

^#^ All variables with *p* ≤ 0.05 was included in the multivariate.

* Statistically significant at *p* ≤ 0.05.

**Fig. 1:**
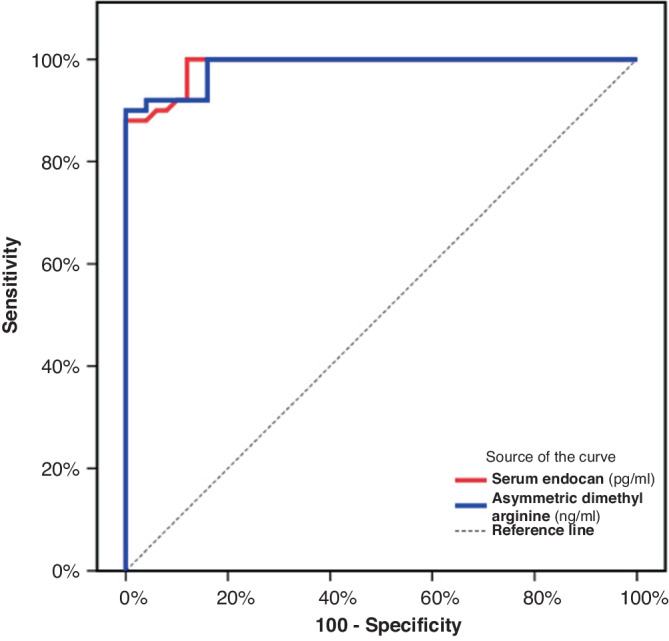
ROC curve for serum endocan and asymmetric dimethyl arginine to discriminate between patients and controls.

ROC curve for serum endocan and ADMA demonstrated a high significance in FMF patients, endocan at a cut-off >80 pg/ml showed 94% sensitivity, 88% specificity, 88.7% positive predictive value (PPV), 93.6% negative predictive value (NPV), and area under the curve (AUC) = 0.988. In addition, ADMA cut-off value >150 ng/ml showed 92% sensitivity, 84% specificity, 85.2% PPV, 91.3% NPV, and AUC = 0.986 Fig. (1).

## Discussion

The present study shows significant elevations in the levels of serum ADMA, endocan, LDL-C, TG, and atherogenic indices (CRI II and AIP) in children with FMF. Additionally, ADMA levels were positively correlated with TC and atherogenic indices (non-HDL-C, CRI I, AIP, and AC).

Endothelium has a vital role in vascular homeostasis. Its functions may be impaired by episodic and chronic systemic inflammations, leading to ED, platelet hyperactivation, and atherosclerotic liability.^13^

Endocan and ADMA are specific vascular endothelium molecules, shown to increase in inflammatory conditions. Endocan has been researched in numerous inflammatory-related cardiometabolic diseases including hypertension, atherosclerotic cardiovascular disease, renal disease, type 2 diabetes mellitus, obesity, and metabolic syndrome in postmenopausal women.^14–17^ In addition, ADMA is involved in inflammation-mediated nitrous oxide depression which stimulates many atherogenic processes such as proinflammatory and chemotactic factors expression, monocyte adhesiveness, and accumulation of oxidatively modified LDL-C in macrophages.^18^

In this study, FMF patients showed significantly elevated circulating levels of serum endocan and ADMA in the attack-free period compared with healthy controls. Few studies investigated the levels of serum endocan and ADMA in FMF pediatric patients and inconsistent results have been reported. According to Ozalper et al. ^5^, serum endocan levels were higher in FMF patients than in healthy controls. Also, a comparable study by Terekeci et al. ^7^, revealed that FMF patients had higher ADMA levels in the acute attack than in the attack-free period and when compared to healthy controls. Conversely, Türkuçar et al. ^14^, documented that there was no difference in serum endocan levels between pediatric FMF patients receiving colchicine medication and healthy controls, suggesting that colchicine may have been effective in reducing inflammation.

It is widely accepted that FMF patients have an increased risk of early vascular changes and atherosclerosis. Several adult studies explained this due to associated persistent inflammation and altered serum lipid profile, and even others demonstrated that FMF-related amyloidosis had a higher CVD risk (probably related to the high ADMA levels especially in the attack-free period) with elevated levels of inflammatory markers, and decreased Flow-mediated dilatation (FMD) measurements reported in those patients.^4,19–22^

Cardiovascular risk increases as the disease advances and the concurrent possible coexistence of dyslipidemia, which is an additional risk factor, contributing to the early onset of atherosclerosis.^19^ Regarding lipid profile, some studies reported a statistically significant lower level of HDL-C between FMF patients and controls not only in the attack-free period, but also in acute attack. Moreover, some demonstrated that even asymptomatic first-degree FMF relatives also had low HDL-C levels. Others demonstrated higher TG levels in FMF patients compared to controls.^14,23,24^ Our study demonstrated that LDL-C and TG were statistically significant higher in patients compared to controls and patients with homozygous mutations showed significantly elevated LDL-C and TG levels compared to those with heterozygous mutations or no mutations.

Moreover, calculated lipid ratios are a more reliable predictor of the progression of atherosclerosis than any other single lipid measure.^25^ In the current study, CRI II and AIP showed a higher statistical significance in patients than in controls. Similarly, Çakırca et al. demonstrated that AIP, AC, and CRI I and II values were significantly higher in FMF patients than in the healthy controls.^4^

Also, Icli et al., reported that FMF patients showed higher AIP values than those of the control group, and there was a positive correlation between carotid intima–media thicknesses, which is used to define preclinical atherosclerosis in FMF patients and AIP values.^20^ Moreover, Vampertzi et al., observed a high statistically significant TG levels and AIP in FMF patients including children and young adults compared to controls, and only a statistically elevated AIP on comparing children subgroup to controls.^26^

Studies in adult patients showed that elevated ADMA levels have been associated with various atherosclerosis risk factors such as ageing, hypercholesterolemia, hypertension, diabetes mellitus, insulin sensitivity and renal failure.^18^ A previous study in children with epilepsy reported a significant positive correlation between ADMA,TG, and LDL-C,^27^ but as far as we know, no other studies showed a relationship between serum ADMA, endocan and lipid profile in FMF pediatric patients. In contrast to endocan, our study showed a positive significant correlation between ADMA and serum total cholesterol, non-HDL cholesterol, CRI I, Atherogenic index of plasma and Atherogenic coefficient in FMF pediatric patients.

The points of strength of our study include that atherogenic indices were calculated in addition to conventional lipid profile parameters for a better assessment of lipid profile and compared to serum endocan and ADMA (in the attack-free period) for evaluation of subclinical inflammation. Moreover, genetic testing was done for all cases.

### Limitations

First, the small number of the studied patients, considering that FMF is a rare disease. Second, single measurement of serum endocan and ADMA levels was done. Third, lacking assessment maneuver for vascular wall status like carotid intimal thickness or FMD. In addition, all FMF patients included in the study were under colchicine treatment, which has contradictory relationship with serum lipids.

Therefore, we urge further research into the role of serum ADMA, endocan, lipid profiles, and atherogenic indices in FMF pediatric patients as potential additional markers for the early diagnosis of atherosclerotic cardiovascular risk.

## Conclusions

Serum endocan and ADMA were elevated in children with FMF during attack-free periods pointing to continuity of endothelial subclinical inflammation. Despite mild changes in lipid profile, atherogenic indices were significantly elevated exhibiting a further risk in these patients. So, it’s important to follow lipid profile and atherogenic indices in these patients especially with elevated levels of ADMA for early detection of cardiovascular risk.

## Data Availability

All datasets presented in this study are included in the article.

## Material availability

Correspondence and requests for materials should be addressed to A.S.A.H.
